# Visualizing nanoscale 3D compositional fluctuation of lithium in advanced lithium-ion battery cathodes

**DOI:** 10.1038/ncomms9014

**Published:** 2015-08-14

**Authors:** A. Devaraj, M. Gu, R. Colby, P. Yan, C. M. Wang, J. M. Zheng, J. Xiao, A. Genc, J. G. Zhang, I. Belharouak, D. Wang, K. Amine, S. Thevuthasan

**Affiliations:** 1 Environmental and Molecular Sciences Laboratory, Pacific Northwest National Laboratory, Richland, Washington 99354, USA; 2 Energy and Environmental Directorate, Pacific Northwest National Laboratory, Richland, Washington 99354, USA; 3 FEI Company, 5350 NE Dawson Creek Dr., Hillsboro, Oregon 97124, USA; 4 Qatar Environment and Energy Research Institute, Qatar Foundation, PO box 5825, Doha, Qatar; 5 Chemical Sciences and Engineering Division, Argonne National Laboratory, Argonne, Illinois 60439, USA

## Abstract

The distribution of cations in Li-ion battery cathodes as a function of cycling is a pivotal characteristic of battery performance. The transition metal cation distribution has been shown to affect cathode performance; however, Li is notoriously challenging to characterize with typical imaging techniques. Here laser-assisted atom probe tomography (APT) is used to map the three-dimensional distribution of Li at a sub-nanometre spatial resolution and correlate it with the distribution of the transition metal cations (*M*) and the oxygen. As-fabricated layered Li_1.2_Ni_0.2_Mn_0.6_O_2_ is shown to have Li-rich Li_2_*M*O_3_ phase regions and Li-depleted Li(Ni_0.5_Mn_0.5_)O_2_ regions. Cycled material has an overall loss of Li in addition to Ni-, Mn- and Li-rich regions. Spinel LiNi_0.5_Mn_1.5_O_4_ is shown to have a uniform distribution of all cations. APT results were compared to energy dispersive spectroscopy mapping with a scanning transmission electron microscope to confirm the transition metal cation distribution.

## Abstract (web summary)

It is challenging to quantitatively diagnose the lithium-ion distribution in batteries. Here, the authors use laser-assisted atom probe tomography to probe the nanoscale compositional fluctuations of lithium ions in two popular lithium-ion battery cathodes before and after electrochemical cycling.

## Graphical abstract

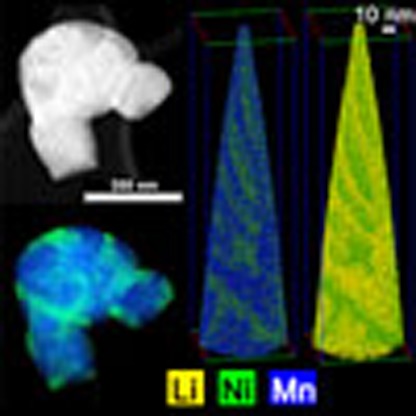

The portable consumer electronics revolution has driven the development of Li-ion batteries for efficient energy storage over the last decade^1^,^2^. Currently, there is also a strong interest in developing cost-effective, rapidly recharging Li-ion batteries suitable for long range electric vehicles^3^. As cathodes constitute a substantial portion of the volume and cost of a battery significant effort has been focused on the development of next-generation cathode materials^4^,^5^. Designing materials that can retain structural integrity after repeated cycling is a substantial challenge^4^. Fast ionic transport during electrochemical cycling of a material depends critically on the initial structure and crystal stability. The presence and stability of channels for fast Li-ion diffusion in cathode materials is an important design criterion for developing next-generation cathode materials for Li-ion batteries with higher capacity and long-term energy storage performance. Understanding nanoscale distribution of all of the elements that makeup Li-ion battery cathodes—especially Li ions—as a function of different synthesis procedures and extents of electrochemical cycling is a critical step towards developing new materials.

Li-rich layered cathode materials with the general formulae Li[Li_1/2-2*x*/3_Ni_*x*_Mn_2/3−*x*/3_]O_2_ where 0<*x*<1/3 and more specifically, Li_1.2_Ni_0.2_Mn_0.6_O_2_—have been demonstrated with capacities >250 mAh g^−1^: significantly higher than the 140-mAh g^−1^ capacity of the best LiCoO_2_ cathodes, used widely in consumer electronics^6^,^7^,^8^,^9^. Structurally layered Li_1.2_Ni_0.2_Mn_0.6_O_2_ is considered to be a phase mixture of the trigonal Li*M*O_2_ (R-3m) and monoclinic Li_2_*M*O_3_ (C2/m ) phases (*M*=Ni, Mn). Both of these structures can be represented as repeating layers of transition metal ions, O and Li. Recently, compositional segregation of Ni to surfaces and grain boundaries within some particles and partitioning of Mn away from Ni-rich regions in layered Li_1.2_Ni_0.2_Mn_0.6_O_2_ has been shown by energy dispersive spectroscopy (EDS) tomography^10^,^11^,^12^. By comparison of high-angle annular dark-field scanning transmission electron microscopy (HAADF–STEM) images to multislice simulations, the Ni-rich regions were shown to be consistent with an R-3m phase and the Ni-deficient regions a C2/m phase^12^. Electron energy-loss spectroscopy (EELS) analysis has indicated a 7:9 Ni:Mn ratio (0.77) in the Ni-rich regions, best matching Li(Ni_0.5_Mn_0.5_)O_2_, and a 5:42 Ni:Mn ratio (0.12) in the Ni-deficient regions, best matching Li_2_MnO_3_ (12). X-ray diffraction from as-prepared Li_1.2_Ni_0.2_Mn_0.6_O_2_ predominantly matched an R-3m structure, with the exception of three peaks between 20 and 25° matching the C2/m structure^8^,^13^,^14^. However, no detailed information was found on the distribution of Li, or of its correlation to other elements in the lattice, within the published literature.

Under battery charge–discharge cycling, layered Li_1.2_Ni_0.2_Mn_0.6_O_2_ cathodes were observed to develop a thin surface reconstruction layer, featuring structural transformation, Mn and Ni enrichment, oxygen vacancy formation and Li depletion^15^,^16^,^17^. Due to the structural and chemical change of the thin surface layer, it is believed to be a likely main contributor to voltage fading^18^. Furthermore, it has been proven that the surface layer continues to grow in thickness during continuous cycling^15^,^19^. EDS analysis of cycled layered Li_1.2_Ni_0.2_Mn_0.6_O_2_ has indicated composition variations even within the thin surface reconstruction layer^15^. To understand the structural degradation mechanism and seek a means to suppress the surface reconstruction, requires monitoring the evolution of all elements quantitatively at different states of cycling. However, despite substantial characterization efforts in previous studies, quantifying lithium depletion and subtle changes in oxygen concentration in the surface layer have been proven to be elusively challenging.

High-voltage spinel LiNi_0.5_Mn_1.5_O_4_, is considered one of the most promising candidates for hybrid electric vehicle batteries^2^,^20^,^21^. Stoichiometric LiNi_0.5_Mn_1.5_O_4_ is known to have a spinel structure with ordered P4_3_32 or disordered 
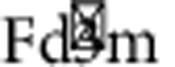
, depending on the post-synthesis annealing temperature^22^,^23^,^24^. This order–disorder phase transformation is expected to occur during higher temperature annealing as a result of generation or elimination of oxygen vacancies, by affecting the presence of Mn^3+^ in the lattice^23^,^24^. The disordered phase of LiNi_0.5_Mn_1.5_O_4_ is shown to have better electrochemical performance than the ordered spinel, owing to its higher electronic conductivity in the presence of increased disordered phase and/or Mn^3+^ concentration^22^,^24^,^25^,^26^,^27^.

The common obstacle to using these materials is capacity and voltage fading, believed to be closely related to a gradual structural evolution, governed by the spatial distribution of Li ions and their correlation with other ions in the lattice. Therefore, one of the great challenges facing the development of these high-voltage cathode materials for Li-ion batteries is to locate the spatial distribution of ions with sub-nanometre-scale spatial resolution. Aberration-corrected scanning and conventional transmission electron microscopy (S/TEM)^28^,^29^,^30^,^31^ and soft X-ray imaging/spectroscopy^32^ have been used to spatially map the transition metal cations within Li-ion battery cathodes, but these techniques do not have sufficient sensitivity to map Li at sub-nanometre scales, especially in three-dimensional (3D). Atom probe tomography (APT) is uniquely capable of providing quantitative 3D, sub-nanometre-scale compositional characterization of oxides and composites^33^,^34^,^35^,^36^,^37^,^38^, with sensitivity for the Li, the O, and the transition metal cations^39^,^40^,^41^,^42^,^43^,^44^,^45^,^46^,^47^,^48^,^49^,^50^ in a Li-ion cathode material. While Li_1.2_Ni_0.2_Mn_0.6_O_2_ is expected to be a two-phase mixture, LiNi_0.5_Mn_1.5_O_4_ is expected to have a uniform cation distribution, providing a baseline evaluation of the ability of APT in unambiguously analysing the Li distribution in Li-ion battery cathode materials.

With this goal in mind, we provide here the first evidence for achieving unambiguous 3D sub-nanometre-scale spatially resolved mapping of Li, Ni, Mn and O in two categories of the advanced Li-ion battery cathode materials: layered-Li[Li_0.2_Ni_0.2_Mn_0.6_]O_2_ and spinel LiNi_0.5_Mn_1.5_O_4_, using APT. APT results from as-fabricated and cycled layered-Li[Li_0.2_Ni_0.2_Mn_0.6_]O_2_ are compared with obtain insights towards mechanisms for cycling dependent capacity loss.

## Results

### Element distribution in as-fabricated Li_1.2_Ni_0.2_Mn_0.6_O_2_

**Figure 1:**
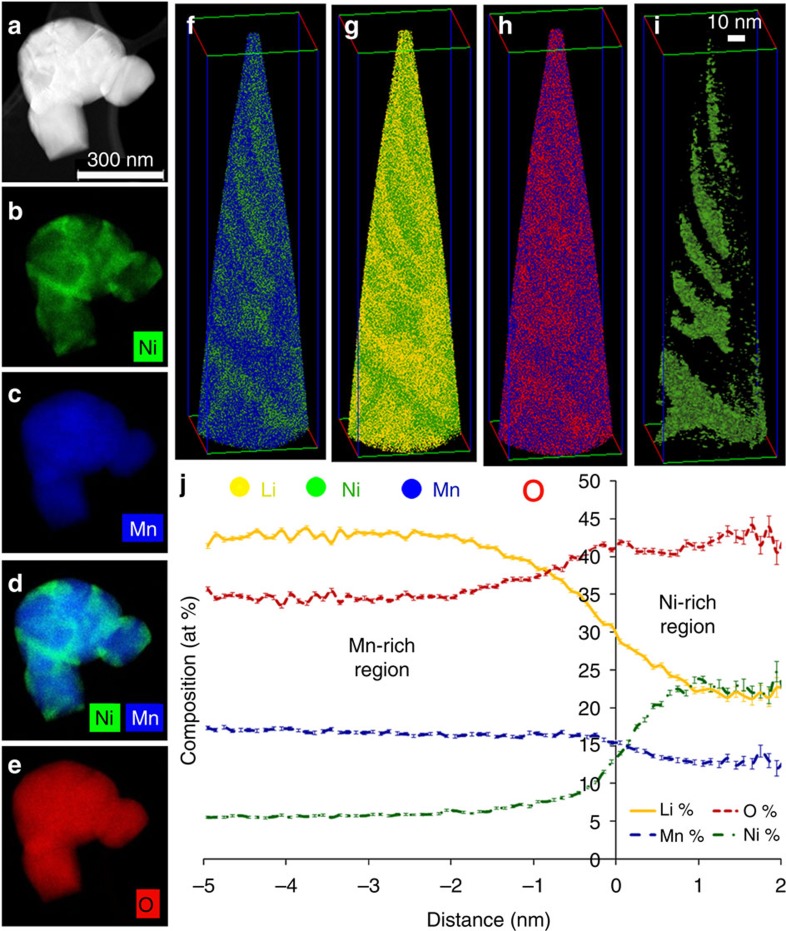
Comparison of TEM EDS tomography with APT results of layered-LNMO. (**a**) STEM image and EDS maps of (**b**) Ni (green) (**c**) Mn (blue) (**d**) Ni and Mn composite map (**e**) O (red). Two element composite maps of the APT reconstructions are shown from **f** to **h** where each dot correspond to an atom in the reconstruction. (**f**) Mn (blue) and Ni (green) ion distribution (**g**) Li (yellow) and Ni (green) ion distribution and (**h**) O (red) and Mn (blue) ion distribution. (**i**) 13 at % Ni isocomposition surface highlighting the Ni-rich regions in the reconstruction (**j**) Proximity histogram^51^ obtained across the 13 at% Ni isocomposition surface showing concentration partitioning between Ni-rich and Mn-rich regions.

Before APT analysis, the layered-Li[Li_0.2_Ni_0.2_Mn_0.6_]O_2_ and spinel LiNi_0.5_Mn_1.5_O_4_ particles—henceforth, layered-LNMO and spinel-LNMO—were analysed using STEM imaging and EDS mapping. An annular dark-field STEM image and EDS maps of a typical layered-LNMO particle are shown in Fig. 1a–e providing a clear evidence of non-uniform distribution of Ni and Mn within a nanoparticle. A layered-LNMO nanoparticle was subsequently analysed with APT using 20-pJ laser pulse energy to image and quantify Li, Ni, Mn and O distributions. In the atom-probe mass-to-charge spectra of layered-LNMO, elemental peaks of Li^1+^, Ni^1+^,Ni^2+^, Mn^1+^,Mn^2+^ and O^1+^ are visible, in addition to molecular species peaks for O_2_^1+^, MnO^1+^,MnO^2+^, MnO_2_^1+^,MnO_2_^2+^, MnO_3_^1+^, MnO_3_^2+^, MnO_4_^1+^, Mn_2_O^2+^,Mn_2_O_2_^1+^, Mn_2_O_3_^1+^, Mn_2_O_4_^1+^, Mn_2_O_5_^1+^, Mn_2_O_6_^1+^, NiO^1+^, NiO^2+^, NiO_2_^1+^, NiO2^2+^, Ni_2_O^2+^, NiO_3_^1+^, MnNiO^2+^, MnNiO_2_^1+^, MnNiO_3_^1+^ and MnNiO_4_^1+^ (Supplementary Fig. 1). Of the total ion counts, 80.12% corresponded to elemental evaporation of Li, Ni, Mn and O. Out of all the molecular species evaporated, O_2_, MnO, NiO, MnO_2_ and MnO_3_ combined constituted 18.37% of the total ion counts. All remaining complex ions corresponded to only 1.51% of the total ion counts. Such complex molecular species evaporation is commonly observed during laser-assisted APT analysis of other metal oxides, such as for MgO (ref. 33). The measured concentrations from the APT reconstruction were 41.61±0.03 at% Li, 6.13±0.01 at % Ni, 16.60±0.02 at % Mn, and 35.66±0.03 at % O. The expected atomic concentrations are 30 at % Li, 5 at % Ni, 15 at % Mn, and 50 at % O.

Oxygen is known to be deficient during laser-assisted APT analysis of oxides except at extremely low laser energies^33^. To verify the accuracy of the APT quantification, the measured concentrations of Li, Mn and Ni were renormalized independently of O and compared with the expected composition. In stoichiometric Li_1.2_Ni_0.2_Mn_0.6_O_2_, the Li fraction of the total cations, Li/(Li+Mn+Ni), should be 0.6; Mn, 0.3; and Ni, 0.1. The measured cation fractions were 0.647, 0.258 and 0.095, respectively. The concentration these specimens will be a function of the volume fraction of Ni-rich and Mn-rich regions sampled within the reconstruction, which may account for the minor variation from the expected stoichiometry. The APT reconstruction from layered-LNMO is shown in Fig. 1f–i). The non-uniform spatial distribution of Mn (blue) and Ni (green) ions shown in Fig. 1f is comparable to EDS maps shown in Fig. 1b–d. The ion map showing Li (yellow) with Ni (green) highlights the similarity of the spatial distribution of Li and Mn. Both Li and Mn segregate away from regions of Ni enrichment (Fig. 1g). The O (red) and Mn (blue) ion map shows O segregating to regions with Ni enrichment (Fig. 1h). A 13 at % Ni isocomposition surface image, shown in Fig. 1i, highlights regions enriched in Ni distributed throughout the reconstructed volume. The compositional partitioning across the Ni- and Mn-rich regions was quantified using a proximity histogram calculated perpendicular to the 13 at % Ni isocomposition surface^51^ (Fig. 1j). From the steady-state regions on either side of the interface in proximity histogram, the concentration of Ni-rich region was estimated to be 21.92±0.83 at% Li, 12.89±0.67 at% Mn, 22.65±0.84 at % Ni and 42.53±0.99 at % O and the Mn-rich regions had a concentration of 42.94±0.29 at % Li, 16.91±0.22 at % Mn, 5.69±0.14 at % Ni and 34.36±0.28 at % O. The Ni:Mn ratios in the Ni-rich region and Mn-rich region correspond to 1.76 and 0.34, respectively. These Ni:Mn ratios are considerably higher than those previously obtained from Mn- or Ni-rich regions with STEM-EELS measurements, but two-dimensional (2D) STEM-based maps are measured in projection and thus an average of the composition through the thickness of the LMNO particle. The truly 3D measurements made by APT would therefore be expected to yield a higher maximum Ni:Mn ratio in 3D Ni- or Mn-rich regions.

**Figure 2:**
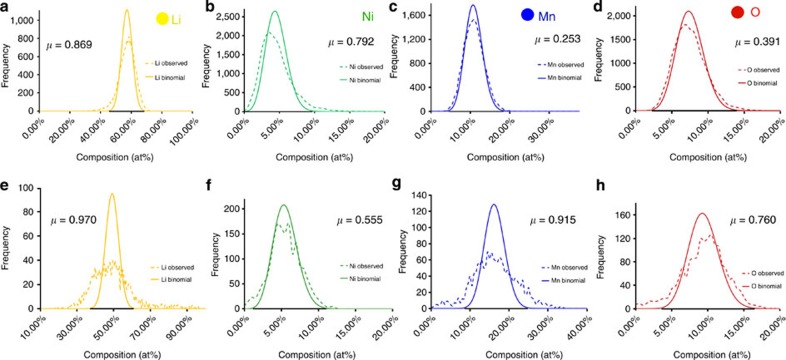
Statistical analysis of element segregation in layered-LNMO. Frequency histogram of as-fabricated layered-LNMO (**a**) Li, (**b**) Ni, (**c**) Mn and (**d**) O with corresponding Pearson coefficients (*μ*) compared with frequency histogram of cycled layered-LNMO (**e**) Li, (**f**) Ni (**g**) Mn, (**h**) O indicating a high degree of non-uniformity in distribution of Li, Ni, Mn and O in both as-fabricated and cycled Li_1.2_Ni_0.2_Mn_0.6_O_2_.

To quantify the extent of phase separation, frequency distribution analysis was performed on the APT results. The entire APT data set was divided in-to 200-atom bins, the composition of each bin was calculated and a histogram was plotted. For a random solid solution, a binomial frequency distribution is expected. Any deviation from randomness will lead to a deviation from binomial distribution. The frequency distribution analysis of Li, Ni, Mn and O in layered-LNMO is shown in Fig. 2a–d. Pearson coefficient test can be used to measure the statistical relevance of the observed deviation from randomness^52^,^53^,^54^. Pearson coefficients tending towards 1 indicate statistically relevant extent of compositional segregation^52^,^55^. The Pearson coefficients for elemental Li and Ni obtained from the 200-atom bin size frequency distribution analysis were 0.869 and 0.792, respectively; Mn and O had Pearson coefficient of 0.253 and 0.391, respectively. The NiO, MnO_3_ and O_2_ also had higher Pearson coefficient values of 0.535, 0.201 and 0.129, respectively, with all other molecular species below 0.1. Also all these elemental and molecular species with >0.1 Pearson coefficients resulted in *P* values <0.001 at 95% confidence level during the null-hypothesis testing. These higher Pearson coefficient values and very low *P* values reaffirm the existence of statistically significant extent of compositional segregation in as-fabricated layered-LNMO.

### Li, Ni, Mn and O distribution in Cycled layered-LNMO

On charge–discharge cycling of layered-LNMO cathodes with pre-existing Ni-rich regions, an additional Ni-rich surface reconstruction layer (SRL) has been found to form and grow as a function of cycling^15^,^16^,^17^,^18^. By detailed STEM, EDS and EELS measurements, a compositional partitioning of Ni and Mn has been observed within the SRL. As the Li concentration is expected to vary between the SRL and bulk of the cathode nanoparticle as a function of cycling, layered-LNMO cathodes cycled for 45 cycles were examined by APT. Needle-shaped APT specimens were fabricated from four different cycled layered-LNMO particles and during the needle preparation the needle specimen apex was kept very close to the top surface of the particles. The overall composition for each of the APT results from the four cycled layered-LNMO particles and two as-fabricated layered-LNMO particles are given below in Table 1.

**Figure 3:**
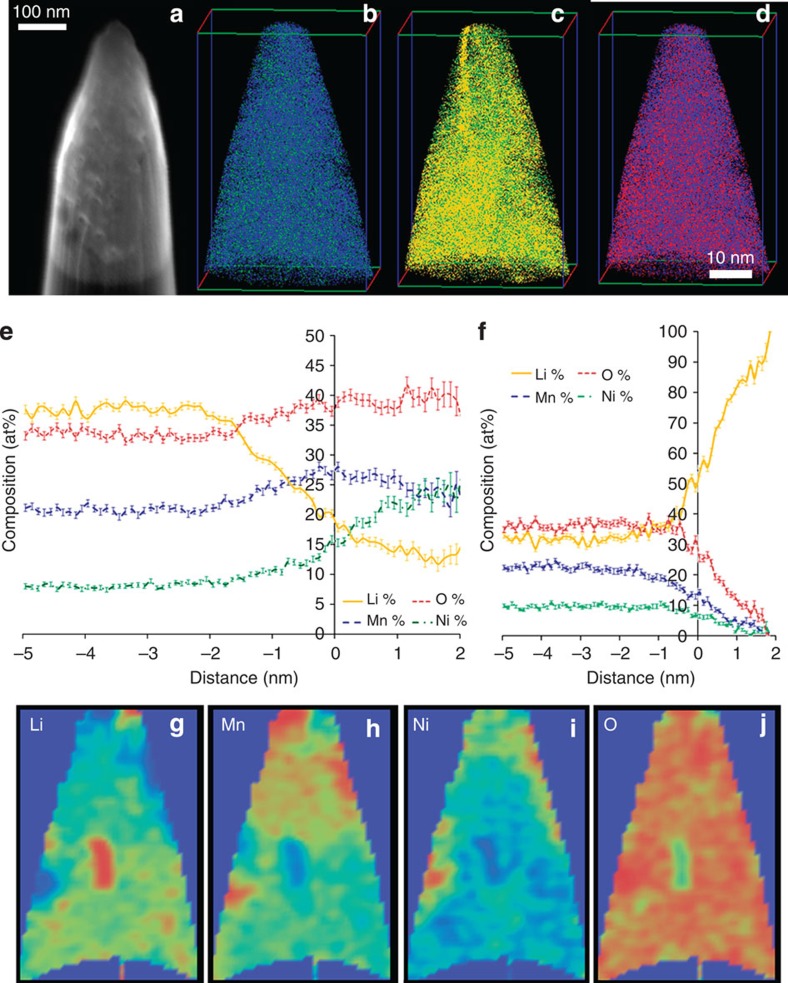
APT results of cycled layered–LNMO. (**a**) The SEM image of the needle specimen of the cycled layered–LNMO (**b**–**d**) ion map of Mn and Ni, Ni and Li, Mn and O respectively. (**e**) Proximity histogram across a 14 at % Ni isocomposition surface and (**f**) proximity histogram across a Li-rich isocomposition surface. (**g**–**j**) 2D composition maps of of Li, Mn, Ni and O for a 5-nm thick slice through a Li-rich inclusion.

A reduction in Li concentration and increase in the Mn and Ni concentration is observed in the cycled layered-LNMO specimens, in agreement with previous literature postulating a loss of Li as a function of cycling^15^,^56^. Two-dimensional composition plots of Li, Ni, Mn and O, as shown in Fig. 3d–g, show that Li, Ni and Mn partially segregate. A proximity histogram plotted across a Ni 14 at% isocomposition surface, shown in Fig. 3e, illustrates the interface between the Ni-rich and Ni-depleted regions within the reconstruction. The proxygram highlights that Li is depleted where Ni is enriched, and that Mn is enriched at the interface between Ni- and Li-rich regions. A second proximity histogram across a Li-rich region indicates a very high Li concentration reaching nearly 100 at %. This apparently facetted region may correspond to a void formed inside the cathode nanoparticle during cycling, with an accumulation of Li. Comparison of the frequency histograms for Li, Mn, Ni and O in the cycled and as-fabricated layered-LNMO indicates an increased phase separation of Li, Mn and O (as indicated by the higher Pearson coefficient for Li, Mn and O of 0.970, 0.915, 0.76, respectively, shown in Fig. 2e–h). The Pearson coefficient of Ni was observed to be 0.555, indicating Ni enrichment near the surface of cycled cathode materials. It is to be noted that recent STEM imaging and EDS measurements observed similar Ni enrichment in as-fabricated and cycled layered-LNMO particles . However, the pre-existing Ni-rich regions in the as-fabricated specimens were not captured within the APT reconstructions from the four cycled layered-LNMO particles. Pearson coefficient of molecular species of NiO (0.523), MnO (0.281), MnO_2_ (0.259), MnO_3_ (0.353), Ni_2_O (0.331), O_2_ (0.218) and NiO_3_ (0.165) were also above 0.1. Each elemental and molecular species with a >0.1 Pearson coefficient values also showed a *P* value <0.001 at 95% confidence interval during null-hypothesis testing, establishing statistically significant segregation of these species in the cycled layered-LNMO.

### Analysis of spinel-LNMO for technique validation

**Figure 4:**
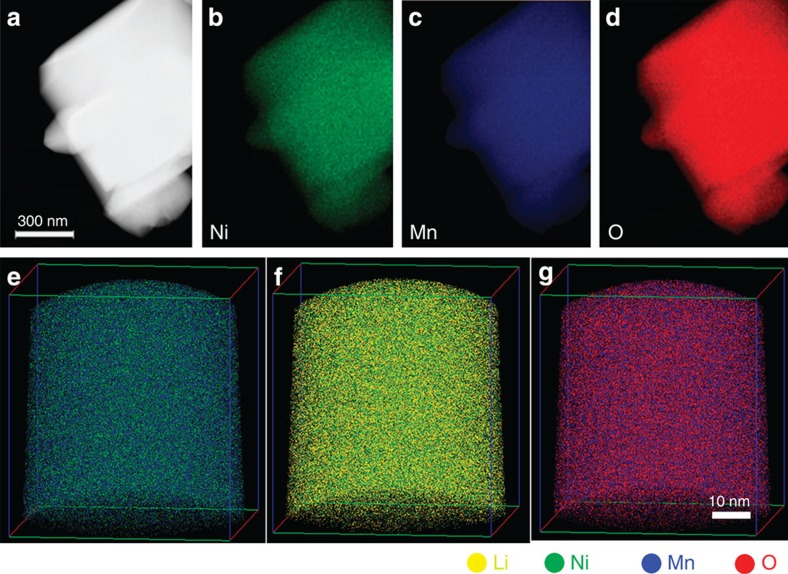
Comparison of TEM EDS tomography with APT results of spinel-LNMO: TEM EDS mapping (**a**–**d**) and APT results (**e**–**g**) for LiNi_0.5_Mn_1.5_O_4_ showing uniform distribution of all elements.

In contrast with layered-LNMO specimens, STEM–EDS mapping of spinel-LNMO indicates a uniform distribution of Mn, Ni and O, as shown in Fig. 4a–d. A mass-to-charge spectra from APT analysis of spinel-LNMO, provided in Supplementary Fig. 1, indicates a distribution of elemental and molecular ions similar to the as-fabricated layered-LNMO. Elemental ions of Li, Mn, Ni and O correspond to 74.23% of the ions detected; 24.2% of the ions detected correspond to O_2_, MnO, NiO, MnO_2_ and MnO_3_ combined, and the remaining 1.58% includes all other complex molecular ions. APT reconstructions of the spinel-LNMO indicated uniform distributions of Li, Ni, Mn and O, as shown in Fig. 4e–g, in agreement with the STEM–EDS results for Ni, Mn and O. The APT-measured composition of spinel-LNMO was 20.06±0.03 at % Li, 9.59±0.02 at % Ni, 27.28±0.03 at % Mn and 43.06±0.04 at % O. The expected composition of these spinel-LNMO specimens is 14.29 at % Li, 7.14 at % Ni, 21.43 at % Mn and 57.14 at % O. As it is expected that oxygen will be underestimated with the 20-pJ laser energy, it is more sensible to renormalize the Li, Ni and Mn ratios excluding oxygen, for example, as *M*/(Li+Mn+Ni). Cation ratios estimated in this manner were 0.35 for Li, 0.48 for Mn, and 0.17 for Ni. The expected cation ratios for stoichiometric LiNi_0.5_Mn_1.5_O_4_ are 0.33 for Li, 0.5 for Mn, and 0.17 for Ni, in excellent agreement with APT results, reinforcing the accuracy of APT in measuring Li, Ni and Mn in these samples.

**Figure 5:**
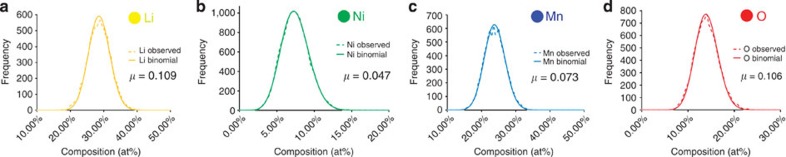
Statistical analysis of element segregation in spinel-LNMO: Frequency distributions of Li, Ni, Mn and O in spinel-LNMO with corresponding Pearson coefficients (*μ*). (**a**–**d**) showing distributions rather close to uniform for Li, Ni, Mn, and O.

Frequency distribution analysis of the APT results from spinel LNMO was conducted to quantify the uniformity of the distribution of each element. Figure 5a–d shows the observed frequency distributions of Li, Ni, Mn and O compared with a binomial distribution. It is clear that spinel-LNMO has a more uniform distribution compared to the as-fabricated layered-LNMO shown in Fig. 2. The Pearson coefficients estimated from the 200 atom bin size frequency distribution analysis for Li, Ni, Mn and O were 0.109, 0.047, 0.073 and 0.106 respectively (shown as inset in Fig. 5), all indicating a very close to uniform distribution. The Pearson coefficient estimated for all the molecular species were also well below 0.1. A Pearson coefficient value close to 0 is indicative of a uniform distribution of elements^52^,^55^. The *P* values estimated at a 95% confidence interval for Li and O was observed to be below 0.001 and the *P* value for Mn and Ni were 0.095 and 0.568. These *P* values in combination with the Pearson coefficient values when compared with layered-LNMO results indicate that there is only a minor deviation from random distribution for Li and O, but Ni and Mn are distributed rather uniformly in the spinel-LNMO APT result.

## Discussion

The Li segregation to Mn-rich, Ni-depleted regions in as-fabricated layered-LNMO suggests a Li_2_MnO_3_ phase with only minor Ni. The Ni-enriched regions with lower Li concentration can be attributed to a Li(Ni_0.5_Mn_0.5_)O_2_ phase. These APT results are consistent with STEM–EDS results from the same materials^11^. The APT results consistent with Li_1.2_Ni_0.2_Mn_0.6_O_2_ being a nanoscale composite mixture of Li*M*O_2_ (R-3m) with Li_2_*M*O_3_ (C2/m) having varying amounts of Li, Mn, and Ni in each of these phases. Partitioning of Li, Ni, and Mn can lead to variation of local electrochemical properties. Recently, it was demonstrated that the capacity fading in layered-LNMO is closely related to the Ni distribution^57^, but those results lacked information about the Li distribution. It is plausible that the local depletion of Li leads to partial deactivation of a particle with cycling, which may contribute to the capacity and voltage fading. APT results from the cycled LNMO provide evidence for Li loss. Partial segregation of Li, Mn and Ni to different regions in the cycled layered-LNMO is shown along with an increased extent of phase separation of Li, Mn and O.

Prior high spatial resolution studies of Li-ion cathode materials have similarly been left to infer the behaviour of Li by studying the changes in the local crystallography and the transition metal cation concentration. This study conclusively demonstrates that laser-assisted APT can be used to not only quantify the Ni and Mn composition in 3D but also the Li, and with sufficient accuracy to postulate the phase. There do not appear to be any barriers to studying the spatial distribution of Li for different synthesis methods, common cathode materials and for varying extents of electrochemical cycling of the cathode material. Quantifying the Li distribution by APT can impact the optimization of cathode synthesis procedures to achieve the highest performance, provide key insights toward the atomic-scale mechanism of capacity decay as a function of cycling, and aid in the effort to create novel Li-ion materials with prolonged lifetimes.

In summary, by comparing the as-fabricated and cycled layered-LNMO we have demonstrated a cycling-induced increased segregation of Li, Mn and O. The APT results of cycled layered-LNMO represent one of the first instances of direct evidence for Li loss in cycled cathode materials, consistent with previous TEM studies and typical explanations for irreversible capacity loss upon cycling of layered cathode materials. Comparison with compositionally uniform spinel-LNMO unambiguously establishes that the laser-assisted APT can differentiate Li segregation in battery-relevant materials at sub-nanometre-scale, in 3D. We anticipate significant application of APT analysis for understanding elemental distribution not just in the as-fabricated cathode materials, but also in electrochemically cycled materials to obtain important insight towards understanding capacity degradation in the cathode materials as a function of extent of cycling.

## Methods

### Material synthesis

Li_1.2_Ni_0.2_Mn_0.6_O_2_ was synthesized by wet chemical process as described briefly here. Nickel sulfate hexahydrate (NiSO_4_·6H_2_O), manganese sulfate monohydrate (MnSO_4_·H_2_O), sodium hydroxide (NaOH), and ammonium hydroxide (NH_3_·H_2_O) were used as the starting materials to prepare Ni_0.25_Mn_0.75_(OH)_2_ precursor. The precursor material was washed with hot water to remove residual sodium and sulfuric species, then filtered and dried inside a vacuum oven set at 80 °C for 24 h. Ni_0.25_Mn_0.75_(OH)_2_ was mixed well with Li_2_CO_3_ and then calcined at 900 C for 15 h to form the cathode materials. Detailed experimental set-up for the synthesis of the materials was reported in Wang *et al.*^58^. A facile solid-state reaction method, which is easy to scale up for mass production, was adopted to synthesize the spinel LiNi_0.5_Mn_1.5_O_4_. In detail, LiNi_0.5_Mn_1.5_O_4_ was prepared by ball milling a mixture of Li_2_CO_3_, NiO and MnCO_3_ (all from Aldrich) in stoichiometric amount for 4 h followed by calcination at 900 °C for 24 h in air with the heating rate of 10 °C min^−1^ and cooling rate of 5 °C min^−1^.

### Electrochemical cycling

The electrochemical cycling of layered-LNMO particles were conducted using coin cells configuration with metallic Li as counter electrode, separator of Celgard K1640 monolayer polyethylene membrane, with 1:2 volume ratio, 1 M Lithium hexafluorophosphate (LiPF6) dissolved in ethyl carbonate and Dimethyl carbonate (DMC) electrolyte in an argon-filled MBraun glovebox. The 45-cycle sample studied in this work was cycled at a rate of 0.1 C between 2.0–4.7 V versus Li/Li^+^ at room temperature. The first cycle charge/discharge profile and ‘charge and discharge' capacities as function of cycle numbers at 2.0–4.7 V versus Li/Li+ are given in Supplementary Fig. 2. The cycled coin cells were disassembled and the cycled electrode was immersed in DMC for 12 h followed by washing by DMC for three times. The washed electrodes were dried in vacuum for 12 h. The cathode material was removed from the Al-foil and grounded to fine powders and deposited on a lacey carbon TEM grid for TEM imaging. The nanoparticles for APT specimens were lifted out from the TEM grid using lift-out method described below.

### STEM/EDS mapping

The STEM and EDS mapping was performed using an FEI Tecnai Osiris microscope at 200 kV. The samples were dispersed onto a holey-carbon TEM grid and imaged accordingly. The FEI Tecnai Osiris microscope is equipped with a state-of-the-art Super-X EDS detector system, which allows efficient and fast mapping at nanoscale.

### Atom probe specimen preparation

Needle-shaped specimens for APT analysis were prepared by lift-out procedure using an FEI Helios 600 Nanolab focused-ion-beam/scanning electron microscope (FIB/SEM). Li_1.2_Ni_0.2_Mn_0.6_O_2_ nanoparticles were dispersed on a Si substrate; individual nanoparticles were lifted out by contact with OmniProbe nanomanipulator and transferred onto a Si microtip array. Once the nanoparticles were placed on top of Si microtips, electron-beam-assisted Pt deposition was used to coat the individual nanoparticles. All manipulation of nanoparticles and Pt deposition were done using only the electron beam without Ga-ion beam imaging. After Pt deposition, the nanoparticles were subjected to annular milling using Ga-ion beam to form the final needle specimens of the nanoparticles attached to the Si microtip array. If cavities were observed between a nanoparticle and the Si microtip, electron-beam-assisted Pt deposition was also performed during annular milling. Initial annular milling was conducted at 30 kV and final milling was performed using 2 kV to minimize Ga contamination in the final needle specimen. A schematic of the specimen preparation method is given in Supplementary Fig. 3.

### Atom probe tomography

Laser-assisted APT analysis was conducted using a CAMECA LEAP4000 × HR atom probe tomography system with a 355-nm ultraviolet laser, 20-pJ laser pulse energy, 40 K specimen temperature and evaporation rate maintained at 0.005 atoms per pulse. APT data were reconstructed and analysed using IVAS 3.6.6 software.

## Additional information

**How to cite this article:** Devaraj, A. *et al.* Visualizing nanoscale 3D compositional fluctuation of lithium in advanced lithium-ion battery cathodes. *Nat. Commun.* 6:8014 doi: 10.1038/ncomms9014 (2015).

## Supplementary Material

Supplementary Information Supplementary Figures 1-3

## Figures and Tables

**Table 1:** Composition of as-fabricated and cycled layered-LNMO.

**Material**	**Particle**	**Concentration (at %**)			
		**Li**	**O**	**Mn**	**Ni**
As-fabricated layered-LNMO	P1	41.61	35.66	16.6	6.13
	P2	40.75	36.25	17.03	5.96
Cycled layered-LNMO after 45 cycles	P1	30.67	35.37	24.4	9.56
	P2	32.59	33.86	25.61	7.94
	P3	34.59	34.6	23.21	7.6
	P4	34.91	34.48	21.38	9.23

The quantified overall composition of 2 different nanoparticles of as-fabricated layered-LNMO and 4 different nanoparticles from cycled layered-LNMO after 45 cycles.
