# Supplementary material for: Visualizing nanoscale 3D compositional fluctuation of lithium in advanced lithium-ion battery cathodes
Source: Nat Commun. 2015 Aug 14;6:8014. doi: 10.1038/ncomms9014 (PMC4557343; doi:10.1038/ncomms9014)
Supplement: Supplementary Information — Supplementary Figures 1-3 [file ncomms9014-s1.pdf]

Supplementary Figures

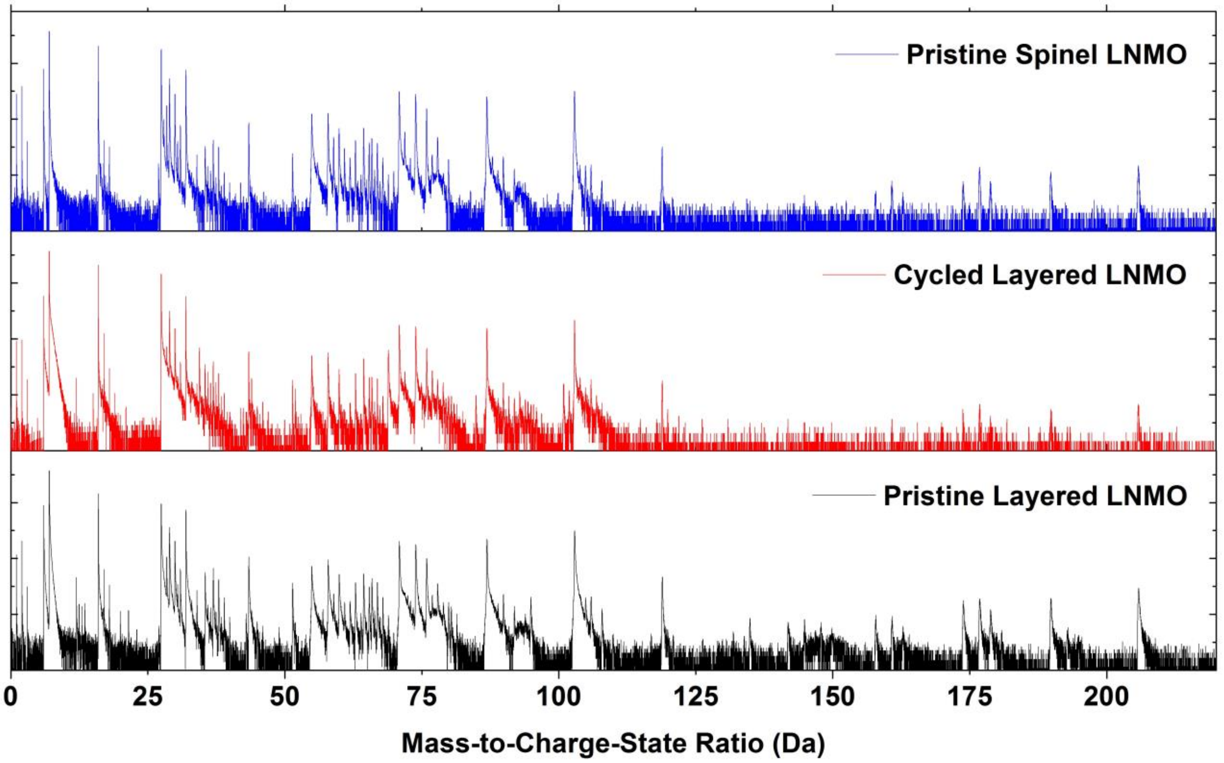

Supplementary Figure 1: mass-to-charge spectra comparison. Mass-to-charge spectra from $Li_{1.2}Ni_{0.2}Mn_{0.6}O_2$ and $LiNi_{0.5}Mn_{1.5}O_4$ analyzed using 20 pJ pulse energy in CAMECA LEAP4000XHR atom probe tomography system.

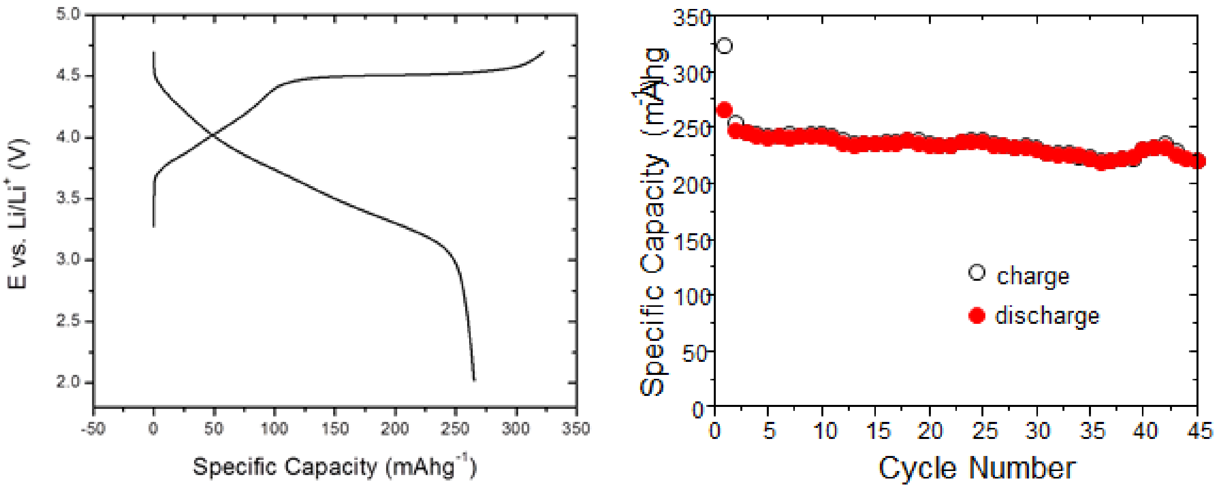

Supplementary Figure 2: electrochemical cycling results. (a) First cycle charge/discharge profile. (b) charge and discharge capacities as function of cycle numbers at 2.0-4.7V vs. Li/Li^+.

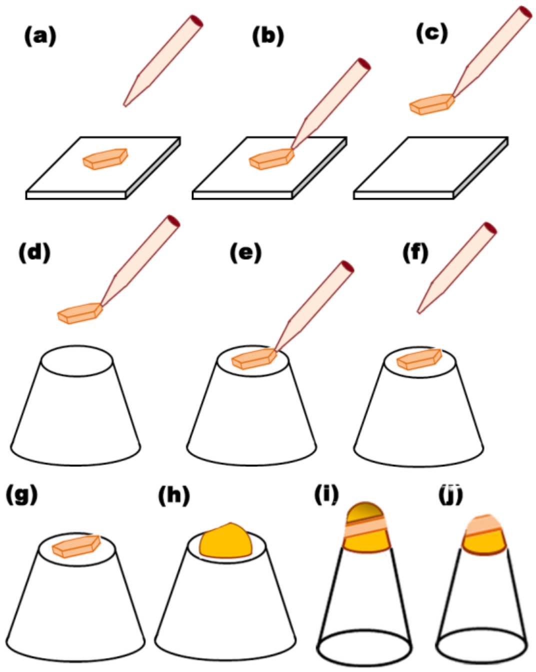

Supplementary Figure 3: Schematic images for APT needle specimen preparation. The procedure start by (a) depositing the nanoparticles on a flat Silicon wafer and loading in a focused ion beam scanning electron microscope equipped with an omniprobe nanomanipulator shown above the sample in (a). Then using only the electron beam image, the omniprobe nanomanipulator is lowered and contacted to a nanoparticle, as shown in (b) which result in a temporary electrostatic attachment of the nanoparticle on to the omniprobe. (c) liftout the attached nanoparticle and (d) transfer it to a microtip array post, which are commercially available prefabricated Silicon microposts with about 2 micron end diameter. Then on (e) contacting the omniprobe and attached nanoparticle on top of the Silicon microtip array the nanoparticle attachment to omniprobe can be broken resulting in placing the nanoparticles on top of the microtip array (f, g). Subsequently electron beam assisted platinum deposition is used to deposit a protective Pt layer on the entire nanoparticle (h) which will protect the particle during annular milling. Then annular milling of the specimen is conducted with reducing inner diameter patterns (I, j) to finally result in a needle with a section of the nanoparticle on the top apex of the needle specimen (j). This is specifically showing the process for a flake shaped nanoparticle specimen. For a more equiaxed or spherical particle with close to 100nm diameter diameter, almost the entire particle can be analyzed within a single needle specimen.
